# Predictors of high HIV+ prevalence in Mozambique: A complex samples logistic regression modeling and spatial mapping approaches

**DOI:** 10.1371/journal.pone.0234034

**Published:** 2020-06-04

**Authors:** Jerry John Nutor, Precious Adade Duodu, Pascal Agbadi, Henry Ofori Duah, Kelechi Elizabeth Oladimeji, Kaboni Whitney Gondwe

**Affiliations:** 1 Department of Family Health Care Nursing, School of Nursing, University of California, San Francisco, California, United States of America; 2 East Surrey Hospital, Redhill, England, United Kingdom; 3 Department of Nursing, Faculty of Allied Health Sciences, College of Health Sciences, Kwame Nkrumah University of Science and Technology, Kumasi, Ghana; 4 Research Department, FOCOS Orthopaedic Hospital, Accra, Ghana; 5 Department of Public Health, Faculty of Health Sciences, University of Fort Hare, Eastern Cape, South Africa; 6 College of Nursing, University of Wisconsin-Milwaukee, Milwaukee, Wisconsin, United States of America; University of Pretoria, SOUTH AFRICA

## Abstract

**Introduction:**

The burden of HIV infection in southern Africa is a public health concern with an increasing number of new infections. This study sought to investigate the predictors of HIV prevalence in Mozambique through a complex samples logistic regression and spatial mapping approach using nationally representative data.

**Methods:**

We conducted a secondary data analysis using the 2015 Mozambique Demographic and Health Survey and AIDS Indicator Survey. The analysis performed in four stages while incorporating population survey sampling weights did the following: i) created a complex sample plan file in SPSS, ii) performed the weighted estimate of HIV prevalence, iii) performed complex sample chi-square test of independence, and then iv) performed complex sample logistic regression modeling.

**Results:**

Out of 11,270 participants, 1,469 (13.0%) tested positive for HIV. The prevalence of HIV infection was higher in females (15.1%) than males (10.2%). We found that urban dwellers were more likely to be HIV-positive compared to rural dwellers (AOR: 1.70; CI: 1.27, 2.27). We observed provincial variations in HIV prevalence, with Maputo Cidade (17.4%), Maputo Provincia (22.6%), Gaza (25.2%) recording higher prevalence above the national estimate. Other independent predictors of HIV infection in Mozambique included age, education level, marital status, total lifetime sexual partners, and having had an STI in the last 12 months.

**Conclusions:**

The study revealed associations between high-risk sexual behavior and HIV infection. Results from our spatial mapping approach can help health policy makers to better allocate resources for cost-effective HIV/AIDS interventions. Pre-Exposure Prophylaxis (PrEP) campaigns among high-risk groups should be pursued to lower the reservoir of HIV among high-risk groups.

## Introduction

Globally, the human immunodeficiency virus (HIV) is a leading cause of morbidity and mortality, especially in sub-Saharan Africa [1]. In 2018, about 37.9 million people were living with HIV globally, with a disproportionate 70% living in sub-Saharan Africa [2]. Over the years, effective antiretroviral therapies (ARTs) have evolved, thereby increasing the life expectancy and quality of life of HIV-infected individuals [3–5]. The global community, in its effort to alleviate HIV infections, set an ambitious goal dubbed the “90-90-90 policy” in 2014. [2] This initiative seeks to ensure that by 2020, 90% of people living with HIV are aware of their HIV status, 90% of those diagnosed with HIV have access to ART, and 90% of those receiving ART achieve suppression of viral loads [2]. Such concerted global efforts have shifted the survival with HIV infection from a terminal illness to a chronic condition [2, 4].

In Mozambique, the HIV epidemic persists as a critical public health and economic challenge [6]. Currently, Mozambique is among the top ten countries globally with the highest prevalence of HIV [1]. In 2018, an estimated 2.2 million people were living with HIV, and there were 54,000 reported HIV-related deaths [1]. A disproportionately high incidence has been reported in the Central and Southern Provinces of the country and among adult women aged 15–49 years [7]; thus, HIV prevalence varies by region, sex, and age in Mozambique [8]. Cultural practices among Mozambicans such as cleansing rituals for widows and widowers, which vary from region to region and are gender-dependent on the person subjected to the ritual, have been reported as key to the spread of HIV infections [9]. Previous studies have also reported risk factors for HIV to include multiple sexual partners [10–12], extramarital sex [10], intimate partner violence [13–15], poverty or household wealth [16], and infrequent or lack of condom use [10, 17]. Others include religion, [18] having an untreated sexually transmitted infection such as herpes simplex virus type 2, mobility and migration, [19] and rural-urban residence status [16, 19]. Despite the significant strides made by Mozambique, HIV prevalence remains high with an increasing number of new infections. Therefore, there is a need for cutting-edge research to identify subpopulations and groups that may be at risk to inform effective policy implementation.

One method to achieve this goal is to use statistical and spatial mapping approaches to estimate the highly heterogeneous HIV prevalence and identify its drivers. The addition of a spatial mapping approach to the prominently-featured regression analysis has become necessary because it is important to critically examine spatial heterogeneity and unmask socio-economic groups that are more at risk of the disease [7, 20]. The use of both spatial mapping approaches and logistic regression techniques to estimate HIV prevalence and determine high-risk groups has been heavily documented [21, 22]. Despite the increasing use of spatial heterogeneity in studying HIV prevalence and its determinants, this technique has rarely been employed in Mozambique using recent nationally representative data. Therefore, this study aimed to model the predictors of HIV prevalence in Mozambique through complex samples logistic regression, spatial mapping approaches and disaggregated analysis using the 2015 nationally representative Demographic Health Survey and AIDS Indicator Survey datasets.

By adopting this statistical approach, specific socio-economic groups where HIV infections are concentrated will be identified. This awareness of low-risk and high-risk socioeconomic groups will inform policymakers in designing effective and culturally acceptable surveillance programs, and model resource allocation for implementing evidence-based control strategies [7, 20]. Further, the availability of HIV spatial distribution stratified by key socio-economic variables could inform cost-effective preventive policies and programs aimed at reducing new cases of HIV infections. Ultimately, well-tailored services could be designed to meet the needs of the most affected groups and regions to improve treatment and to ensure non-biased, equitable, and financially sustainable responses [6, 23].

## Materials and methods

### Design and data source and sample

We used an existing dataset from the cross-sectional 2015 Mozambique Demographic and Health Survey (DHS) and AIDS Indicator Survey (AIS) conducted in Mozambique in 2015 [24]. The DHS and AIS employed a multi-stage stratified design. The multistage sampling started with an initial random selection of primary sampling units (PSU) to form the master sample frame for the AIS survey, which was drawn from the sample frame of the 2007 Mozambique General Population and Housing Census. The first stage in the sampling involved the random selection of PSUs, stratified by urban-rural places of residence. In total, 307 PSUs were randomly selected from the master sample frame, with 134 urban PSUs and 173 rural PSUs. Twenty-four (24) households were selected from each PSU, constituting a total household sample size of 7,368 households. Comprehensive information on the sampling design can be found elsewhere [24].

A total of 14,343 individuals were initially identified for sampling. After obtaining and performing Dried Blood Spot specimen testing at the laboratory, final HIV-positive status results were available for 11,270, resulting in an overall response rate of 78.6% [24]. Individual weights were generated for men and women separately, taking into account their response rate in each stratum [25] For the women weights, the household weight was multiplied by the inverse of the individual response rate for women in the stratum [25]. For the men weights, the household weight for the men’s subsample was multiplied by the inverse of the individual response rate for men in the stratum [25].

### Measurement of variables

The dependent variable in these analyses is the HIV/AIDS status, which was measured for each participant included in the survey and was a binary outcome (negative/positive). HIV diagnostic testing was conducted using two rapid tests on a whole blood sample collected from either a finger-prick or venipuncture. More details about the HIV testing and diagnosis algorithm employed for confirmation and tie-breaker in the Mozambique DHS are reported on the DHS website and the final report [24]. The current analysis includes the following independent variables: socio-demographic, biological and behavioral factors. Socio-demographic variables were age (15–19 / 20–24 / 25–29 / 30–34 / 35–39 / 40–44 / 45–49 / 50+), gender (male / female), place of residence (urban / rural), education level (no education / primary / secondary / post-secondary), religion (Catholic /Protestant Christian / Islamic / No religion), marital status (never in union /married / living with partner / widowed / divorced / No longer living together/separated), province (Niassa / Cabo Delgado / Nampula / Zambézia / Tete / Manica / Sofala / Inhambane / Gaza / Maputo Provincia / Maputo Cidade). Behavioral factors include the use of condom/use condom for most recent sex (no / yes / never had sex / not had sex last month), extramarital partner or sexual partners defined as having more than one sexual partner in the past 12 months (yes / no). Biological factors include the presence of a sexually transmitted infection (STI) or its symptoms in the past 12 months (yes/no). Socioeconomic status was assessed using the household wealth index (poorest / poorer / middle / richer / richest). Household wealth index was already estimated and reported in the DHS data. This was created using household characteristics (source of drinking water, type of toilet, sharing of toilet facilities, main material for the roof, walls and floors floor, and type of cooking fuel amongst others household characteristics) and household possessions and assets (ownership of television, radio, vehicle, bicycles, motorcycles, watch, agricultural land, farm animals/livestock, and bank account amongst others). DHS used a principal component analysis (PCA) to assign weights to each asset in each household and cumulative scores were calculated from the assigned weights. Households were ranked according to the cumulative scores from the household assets. The cumulative percentage distribution of the wealth score was estimated and the wealth score values that corresponded to the four cut point values of the quintiles (20th, 40th, 60th, and 80th percentiles) were determined. Households with values less than or equal to the 20th percentile score were assigned poorest, those greater 20% but less than or equal to 40th % were assigned poorer, those greater than the 40th % and less than or equal to the 60th % score were assigned middle, greater than the 60th % and less than or equal to the 80th % score were assigned richer and the richest households were those with scores greater than the 80th percentile score. Wealth index was thus ranked into quintiles: poorest, poorer, middle, richer, and richest [26]. Table 1 presents the fixed format responses for each of these variables.

**Table 1:** Weighted summary statistics of study variables (N = 11,270).

Variables	n* (%)
**HIV/AIDS Status**	
Negative	9801 (87.0)
Positive	1469 (13.0)
**Gender**	
Male	4751 (42.2)
Female	6519 (57.8)
**Age**	
15–19	2279 (20.2)
20–24	1968 (17.5)
25–29	1521 (13.5)
30–34	1293 (11.5)
35–39	1206 (10.7)
40–44	981 (8.7)
45–49	798 (7.1)
50+	1225 (10.9)
**Education**	
No formal education	2363 (21.0)
Primary	6011 (53.3)
Secondary	2673 (23.7)
Post-Secondary	223 (2.0)
**Marital status**	
Never in union	2,393 (21.2)
Married	3,938 (34.9)
Living with partner	3,378 (30.0)
Widowed	463 (4.1)
Divorced	391 (3.5)
No longer living together/separated	707 (6.3)
**Religion**	
Catholic	3368 (29.9)
Protestant Christians	4474 (39.7)
Islam	2216 (19.7)
No religion/others	1205 (10.7)
**Total lifetime sexual partners**	
0	746 (6.6)
1	3195 (28.4)
2	2386 (21.2)
3–4	2320 (20.6)
5–9	1288 (11.4)
10+	709 (6.3)
Undisclosed	620 (5.5)
*Missing*	6
**Use of condom/use condom for most recent sex**	
No	7849 (69.6)
Yes	1327 (11.8)
Never had sex	746 (6.6)
Not had sex last month	1348 (12.0)
**Extra marital/partner sexual partners**	
None	8469 (75.2)
1	2367 (21.0)
2+	430 (3.8)
*Missing*	4
**Had any STI last 12 months**	
No	10813 (96.1)
Yes	442 (3.9)
*Missing*	15
**Household wealth index**	
Poorest	2,123 (18.8)
Poorer	2,148 (19.1)
Middle	2,083 (18.5)
Richer	2,231 (19.8)
Richest	2,684 (23.8)
**Rural/Urban Residence**	
Urban	4145 (36.8)
Rural	7125 (63.2)
**Region**	
Niassa	612 (5.4)
Cabo Delgado	1131 (10.0)
Nampula	2416 (21.4)
Zambézia	1275 (11.3)
Tete	786 (7.0)
Manica	805 (7.1)
Sofala	1072 (9.5)
Inhambane	723 (6.4)
Gaza	913 (8.1)
Maputo Provincia	728 (6.5)
Maputo Cidade	809 (7.2)

*rounded to whole number

### Data analyses

We merged the women’s and men’s demographic characteristics datasets with the dataset containing their final HIV/AIDS test results using variables from the three datasets with unique identifiers. Given that the 2015 Mozambique AIS is a multi-stage stratified design, we adopted a complex sampling design analysis. By choosing this method of analysis, we eliminated the possibility of the underestimating the standard errors associated with the confidence intervals and regression coefficients. Adjusted Odds Ratio (AOR) estimates were reported for the multivariate analysis. The statistical analyses were performed in the Statistical Package for Social Sciences (SPSS) version 21 (IBM Corp, 2012). The analytical steps are reported as follows:

1. We created a complex sample plan file in SPSS using the individual weight, primary sampling unit, and the sample strata for sampling errors variables. The DHS’ computation procedures for generating the sampling errors for each stratum are reported here on page 59 through 69 [24].
2. We performed the weighted estimate of HIV prevalence in Mozambique as well as the weighted summary statistics of the study variables.
3. We performed the complex sample chi-square test of independence to ascertain the relationship between HIV status and the predictor variables.
4. Predictor variables that were statistically associated with the outcome in both the chi-square test of independence and the unadjusted logistic regression model were regressed upon the HIV status variable in a complex sample logistic regression model.

Additionally, we produced spatial maps of the distribution of HIV prevalence per provinces in Mozambique using the Quantum Geographic Information Systems Software version 3.10.0 (QGIS Development Team, 2019). We further stratified the provincial HIV+ prevalence by the study predictor variables to understand the population subgroups that have the highest burden of HIV infection in each province of Mozambique. Given that the sample size information on both HIV+ and HIV- are presented in Tables 1 and 2, we reported only the prevalence (%) of HIV+ in each socioeconomic and demographic subgroups stratified by the province of residence (S1 Table).

**Table 2:** Chi-square test of independence between descriptive variables and the outcome (N = 11,270).

Variables	HIV Status	
	Negative	Positive
	n* (%)	n* (%)
**Gender**		
Male	4266 (89.8)	485 (10.2)
Female	5534 (84.9)	985 (15.1)
χ^2^ = 58.32; p-value = 0.000		
**Age**		
15–19	2182 (95.7)	97 (4.3)
20–24	1771 (90.0)	197 (10.0)
25–29	1290 (84.8)	231 (15.2)
30–34	1052 (81.4)	241 (18.6)
35–39	950 (78.8)	256 (21.2)
40–44	805 (82.1)	176 (17.9)
45–49	674 (84.5)	124 (15.5)
50+	1077 (88.0)	147 (12.0)
χ^2^ = 308.90; p-value = 0.000		
**Education**		
No formal education	2064 (87.4)	298 (12.6)
Primary	5206 (86.6)	806 (13.4)
Secondary	2324 (86.9)	350 (13.1)
Post-Secondary	207 (92.8)	16 (7.2)
χ^2^ = 7.87; p-value = 0.14		
**Marital status**		
Never in union	2227 (93.1)	166 (6.9)
Married	3549 (90.1)	389 (9.9)
Living with partner	2874 (85.1)	504 (14.9)
Widowed	313 (67.6)	150 (32.4)
Divorced	324 (83.0)	67 (17.0)
No longer living together/separated	514 (72.6)	193.53 (27.4)
χ^2^ = 410.37; p-value = 0.000		
**Religion**		
Catholic	3013 (89.5)	355 (10.5)
Protestant Christians	3717 (83.1)	756 (16.9)
Islam	2022 (91.2)	195 (8.8)
No religion/others	1042 (86.5)	162 (13.5)
χ^2^ = 112.94; p-value = 0.000		
**Total lifetime sexual partners**		
0	735 (98.5)	11 (1.5)
1	2939 (92.0)	256 (8.0)
2	2031 (85.1)	355 (14.9)
3–4	1903 (82.0)	417 (18)
5–9	1056 (82.0)	232 (18)
10+	607 (85.6)	102 (14.4)
Undisclosed	525 (84.7)	95 (15.3)
χ^2^ = 247.38; p-value = 0.000		
**Use condom for most recent sex**		
No	6867 (87.5)	982 (12.5)
Yes	1102 (83.1)	225 (16.9)
Never had sex	735 (98.5)	11 (1.5)
Not had sex last month	1096 (81.3)	263 (12.6)
χ^2^ = 20.30; p-value = 0.000		
**Extra marital/union sexual affairs**		
None	7440 (87.8)	1030 (12.2)
1	1978 (83.6)	389 (16.4)
2+	378 (88.1)	51 (11.9)
*Missing*		
χ^2^ = 30.05; p-value = 0.000		
**Had any STI last 12 months**		
No	9449 (87.4)	1364 (12.6)
Yes	336 (76.0)	106 (7.2)
*Missing*		
χ^2^ = 48.28; p-value = 0.000		
**Household wealth index**		
Poorest	1925 (90.7)	198 (9.3)
Poorer	1962 (91.3)	186 (8.7)
Middle	1851 (88.9)	231 (11.1)
Richer	1825 (81.8)	406 (18.2)
Richest	2236 (83.3)	448 (16.7)
χ^2^ = 152.70; p-value = 0.000		
**Rural/urban Residence**		
Urban	3438 (82.9)	707 (17.1)
Rural	6362 (89.3)	763 (10.7)
χ^2^ = 92.89; p-value = 0.000		
**Region**		
Niassa	567 (92.6)	45 (7.4)
Cabo Delgado	980 (86.7)	150 (13.3)
Nampula	2280 (94.4)	136 (5.6)
Zambézia	1087 (85.3)	188 (14.7)
Tete	747 (95.0)	39 (5.0)
Manica	696 (86.5)	109 (13.5)
Sofala	900 (84.0)	172 (16.0)
Inhambane	628 (86.9)	95 (13.1)
Gaza	683 (74.8)	230 (25.2)
Maputo Provincia	564 (77.4)	165 (22.6)
Maputo Cidade	668 (82.6)	141 (17.4)
χ^2^ = 382.90; p-value = 0.000		

*rounded to whole number

### Ethical considerations

The 2015 Mozambique AIS protocol was reviewed and approved by the Ethical Review Committee of the Mozambique Ministry of Health’s National Institute of Health and the Institutional Review Board of ICF International [24]. Informed consent was also obtained from participants before interviewing and collecting blood samples from them [24]. The 2015 Mozambique AIS data is publicly available upon a simple, registration-access request, so we did not seek for further ethical clearance.

## Results

### Sample characteristics

In all, 11,270 participants were included in the analysis. Of this, 1469 (13.0%) tested positive for HIV. Most of the respondents were females (57.8%), were within the age group of 15–19 years (20.2%), with a primary level education (53.3%), were currently married, and were protestant Christians (39.7%). Further, a proportional sum of 64.7% of the respondents reported having had more than one sexual partner in their lifetime, and about 7 out of 10 of the respondents had not used a condom for their most recent sex (69.6%). A quarter of the respondents had had at least one extramarital sexual partner in the last twelve month, and an estimated 3.9% of the respondents had had an STI in the previous 12 months. Many of the respondents belonged to richest households (23.8%), resided in rural areas (63.2%), and resided in the Nampula province (21.4%). Details of descriptive statistics of the study variables are reported in Table 1.

### Chi-square test of independence between descriptive variables and HIV status

A Chi-square test of independence and bivariate logistic regression analyses were performed to ascertain the relationship between the potential predictor variables and HIV status. The results revealed that all the predictor variables considered in the study were significantly associated with HIV status. The proportion of females who were HIV-positive was more than males (15.1% vs. 10.2%), urban higher than rural (17.1% vs. 10.7%) and richer higher than the richest, the middle, the poorer, and the poorest (18.2% vs. 16.7% vs 11.1% vs 8.7% % vs. 9.3%). A detailed description of the chi-square test of association results is presented in Table 2.

### Sociodemographic and behavior factors regressed on HIV status

The adjusted complex samples logistic regression revealed that the following sociodemographic factors were statistically significant predictors of HIV infection in Mozambique: sex, age, education level, marital status, household wealth index, urban/rural residence, and region of residence (Table 3). Compared to males, females were more likely to be HIV+ (AOR: 1.97; CI: 1.63, 2.38). Compared to 15–19 years old persons, the odds of getting infected with HIV was higher in the following age groups: 20–24 years (AOR: 1.58, CI: 1.13, 2.19), 25–29 years (AOR: 2.48 CI: 1.65, 3.72), 30–34 years (AOR: 3.31 CI: 2.32, 4.72), 35–39 years (AOR: 3.88, CI: 2.56, 5.89), 40–44 years (AOR: 3.16, CI: 2.11, 4.72), 45–49 years (AOR: 2.44, CI: 1.59, 3.76), 50+ years (AOR: 1.62, CI: 1.07, 2.45). Compared to persons with higher education, persons with no education (AOR: 2.74, CI: 1.42, 5.26), primary education (AOR: 2.86, CI: 1.49, 5.49), and secondary education (AOR: 2.60, CI: 1.36, 4.99) were more likely to be HIV+. Compared to persons who had never been in intimate union, widowed (AOR: 2.58, CI: 1.75, 3.82), divorced (AOR: 1.68, CI: 1.05, 2.68), and persons who were no longer living together/separated (AOR: 1.88, CI: 1.31, 2.68) were more likely to be HIV+. Compared to persons from the poorest households, persons from the richer households (AOR: 1.43; CI: 1.02, 1.99) were more likely to be HIV+. Urban dwellers were more likely to be HIV+ compared to rural dwellers (AOR: 1.59; CI: 1.17, 2.17). Persons dwelling in Zambézia (AOR: 1.82; CI: 1.06, 3.10), Manica (AOR: 1.99; CI: 1.17, 3.38), Sofala (AOR: 2.03; CI: 1.20, 3.43), Gaza (AOR: 2.76; CI: 1.65, 4.62) or Maputo Provincia (AOR: 2.08; CI: 1.22, 3.54) were more likely to be HIV+ compared to persons in Niassa province.

**Table 3:** Complex sampling logistic regression results of predictors of HIV status (N = 11,270).

Parameters	OR [95% CI for OR]	AOR [95% CI for AOR]
**Gender**		
Male	1	1
Female	1.57 [1.40, 1.75]	1.97 [1.63, 2.38]
**Age**		
15–19	1	1
20–24	2.49 [1.87, 3.32]	1.58 [1.14, 2.19]
25–29	4.00 [2.88, 5.57]	2.48 [1.65, 3.72]
30–34	5.13 [3.86, 6.82]	3.31 [2.32, 4.73]
35–39	6.02 [4.35, 8.32]	3.88 [2.56, 5.89]
40–44	4.89 [3.55, 6.73]	3.16 [2.11, 4.72]
45–49	4.10 [2.90, 5.82]	2.44 [1.59, 3.76]
50+	3.06 [2.21, 4.25]	1.62 [1.07, 2.45]
**Education level**		
Post-secondary	1	1
No formal education	1.87 [1.05, 3.33]	2.74 [1.42, 5.26]
Primary	2.01 [1.13, 3.56]	2.86 [1.49, 5.49]
Secondary	1.95 [1.08, 3.53]	2.60 [1.36, 4.99]
**Marital/Union Status**		
Never in union	1	1
Married	1.47 [1.14, 1.90]	1.12 [0.77, 1.65]
Living with partner	2.36 [1.88, 2.96]	1.16 [0.81, 1.67]
Widowed	6.43 [4.83, 8.55]	2.58 [1.75, 3.82]
Divorced	2.76 [1.86, 4.08]	1.68 [1.05, 2.68]
No longer living together/separated	5.05 [3.89, 6.56]	1.88 [1.31, 2.68]
**Religion**		
Protestant Christians	1	1
None	0.58 [0.46, 0.73]	0.91 [0.73, 1.13]
Catholic	0.47 [0.36, 0.63]	0.76 [0.52, 1.11]
Islam	0.77 [0.63, 0.93]	1.01 [0.83, 1.23]
**Total lifetime sexual partners**		
**1**	1	1
0	0.18 [0.08, 0.37]	0.38 [0.16, 0.90]
2	2.00 [1.68, 2.40]	1.93 [1.61, 2.31]
3–4	2.51 [2.06, 3.06]	2.63 [2.13, 3.26]
5–9	2.52 [1.99, 3.19]	3.73 [2.77, 5.04]
10+	1.92 [1.40, 2.63]	2.78 [1.98, 3.93]
Undisclosed	2.08 [1.48, 2.90]	2.91 [1.98, 4.28]
**Use condom for most recent sex**		
Yes	1	1
No	0.70 [0.60, 0.83]	0.82 [0.68, 1.00]
Never had sex	0.08 [0.04, 0.16]	1.05 [0.76, 1.45]
Not had sex last month	1.13 [0.91, 1.40]	1.05 [0.76, 1.45]
**Extra marital/union sexual affairs**		
None	1	1
One partner	1.42 [1.22, 1.65]	1.05 [0.83, 1.32]
2 partners +	0.98 [0.72, 1.33]	0.67 [0.44, 1.00]
**Had any STI last 12 months**		
No	1	1
Yes	2.19 [1.71, 2.79]	1.74 [1.34, 2.24]
**Household Wealth Index**		
Poorest	1	1
Poorer	0.92 [0.68, 1.25]	0.89 [0.65, 1.22]
Middle	1.21 [0.90, 1.63]	1.09 [0.79, 1.49]
Richer	2.16 [1.64, 2.84]	1.43 [1.02, 1.99]
Richest	1.94 [1.47, 2.56]	1.08 [0.71, 1.64]
**Rural/urban Residence**		
Rural	1	Referent
Urban	1.71 [1.40, 2.10]	1.59 [1.17, 2.17]
**Region**		
Niassa	1	1
Cabo Delgado	1.93 [1.03, 3.64]	1.52 [0.82, 2.83]
Nampula	0.75 [0.46, 1.22]	0.65 [0.39, 1.06]
Zambézia	2.19 [1.31, 3.65]	1.82 [1.06, 3.10]
Tete	0.66 [0.40, 1.10]	0.68 [0.39, 1.17]
Manica	1.97 [1.25, 3.09]	1.99 [1.17, 3.38]
Sofala	2.41 [1.46, 3.99]	2.03 [1.20, 3.43]
Inhambane	1.91 [1.12, 3.26]	1.17 [0.64, 2.13]
Gaza	4.24 [2.79, 6.45]	2.76 [1.65, 4.62]
Maputo Provincia	3.69 [2.31, 5.88]	2.08 [1.22, 3.54]
Maputo Cidade	2.66 [1.71, 4.15]	1.45 [0.84, 2.50]
Strata		21
Units		306
Population size		11239.36
Cox and Snell		0.11
Nagelkerke		0.20
MacFaddan R^2^		0.15

Behaviors that significantly predicted HIV infection included total lifetime sexual partners, having had an STI in the last 12 months, and condom use. The risk of HIV infection increased by the number of lifetime sexual partners reported by the participants; 2 lifetime partners (AOR:1.93; CI: 1.61, 2.31), 3–4 lifetime partners (AOR: 2.63; CI: 2.13, 3.26), 5–9 lifetime partners (AOR: 3.73; CI: 2.77, 5.04), 10+ lifetime partners (AOR: 2.78; CI: 1.98, 3.93), and undisclosed number of partners (AOR: 2.91; CI: 1.98, 4.28). We unexpectedly found that the odds for being tested positive for HIV was significantly lower for individuals who did not use condom compared to those who used condoms in their most recent sex (AOR: 0.82; CI: 0.68, 1.00). Persons with any STI in the last 12 months were more likely to be HIV+ compared to persons without any STI (AOR: 1.74; CI: 1.34, 2.24). The detailed AORs and their corresponding 95% CIs of all the predictors in the model are reported in Table 3.

### Spatial and provincial distribution of HIV prevalence

Using spatio-temporal analysis techniques, we mapped the distribution of HIV prevalence by the province in Mozambique. We further disaggregated provincial HIV prevalence by the socio-demographic, behavioral, biological and SES factors. The specific HIV prevalence of all the variables in each province is presented in the supplementary table (S1 Table).

#### Socio-demographic factors

**Fig 1:**
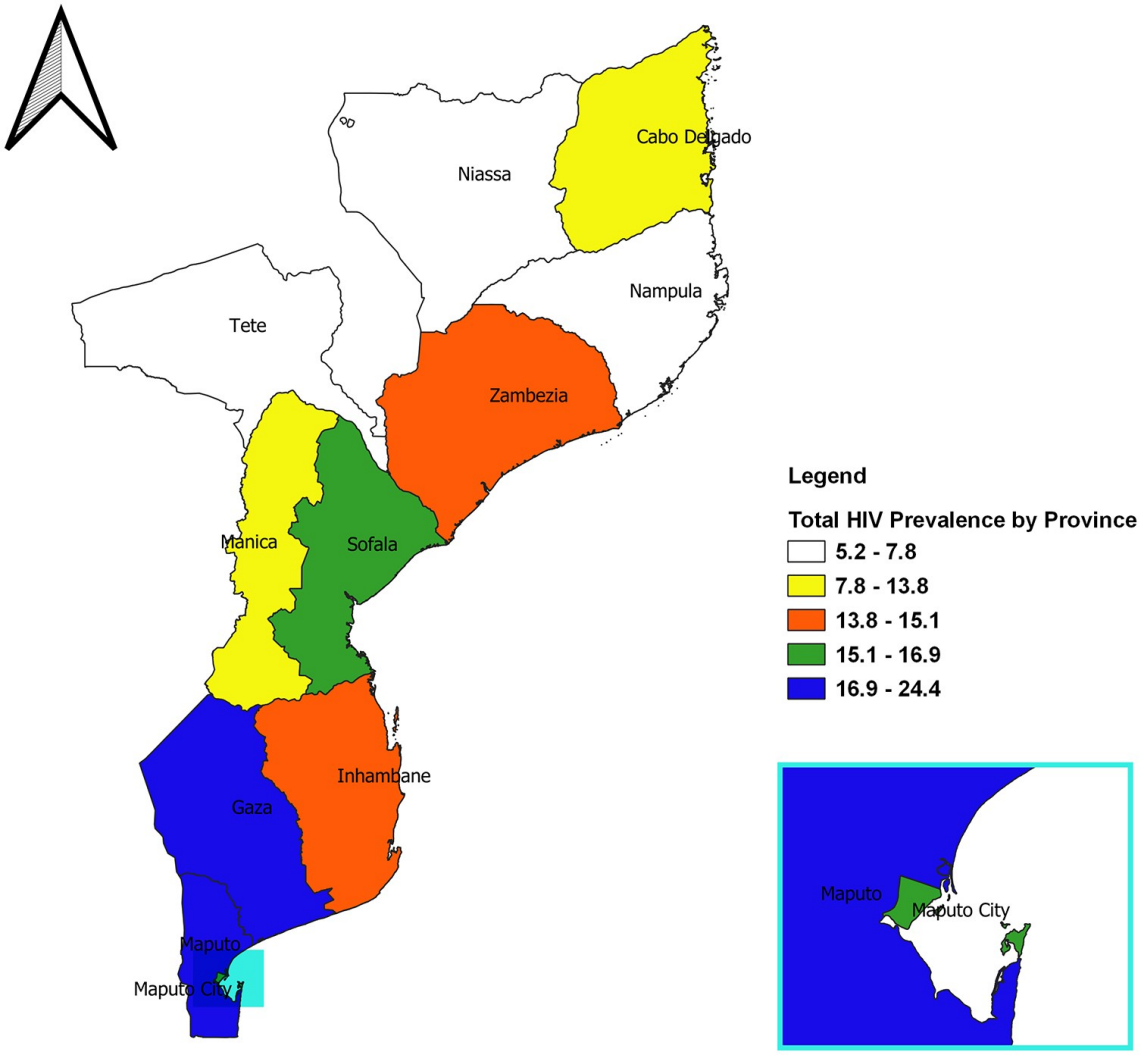
Spatial map of HIV prevalence in Mozambique.

**Fig 2:**
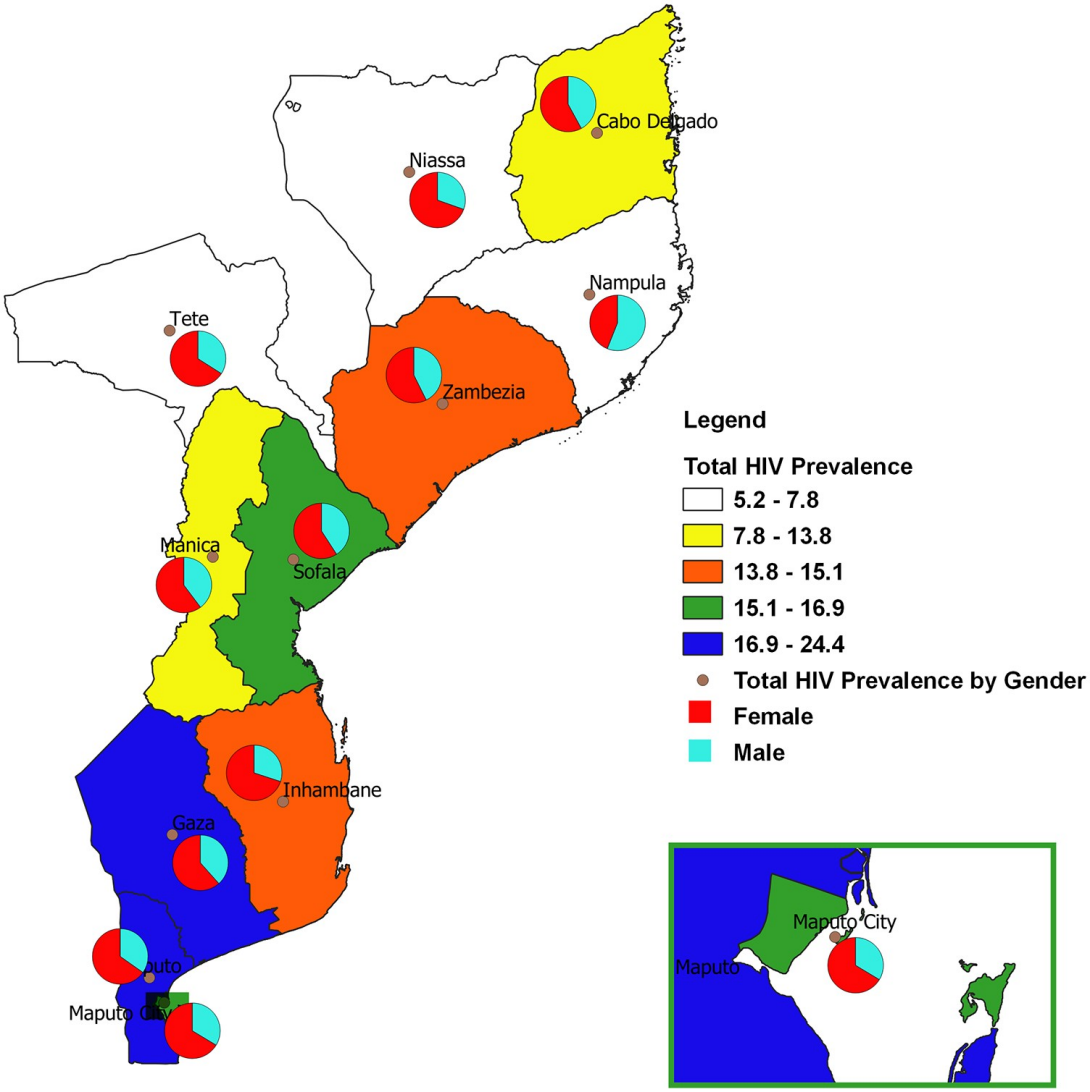
Spatial map of provincial HIV prevalence by gender.

**Fig 3:**
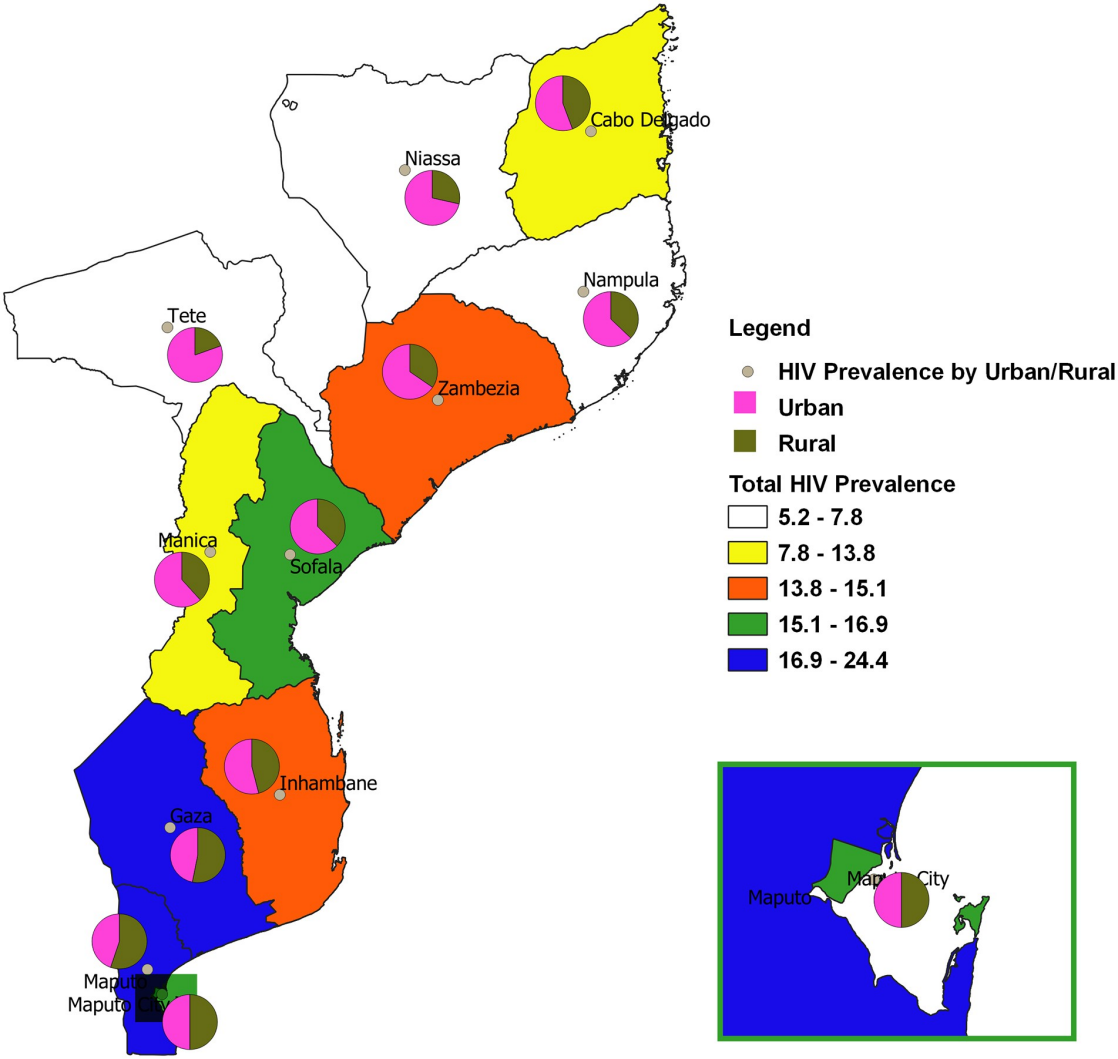
Spatial map of provincial HIV prevalence by place of residence.

Fig 1 showed that the Gaza and Maputo Provincia recorded higher HIV prevalence among the eleven provinces in Mozambique. We found that HIV prevalence among females was higher than males in all the provinces except the Nampula province (Fig 2). Except for Gaza, Maputo, and Maputo Cidade (where the proportion of participants who tested positive for HIV were equal in both urban and rural areas), the remaining 8 provinces recorded more HIV+ cases in urban areas (Fig 3). The majority of the participants who tested HIV+ in Tete, Manica, Maputo Cidade, and Maputo were within the age bracket of 35–39 years (S1 Table). In Gaza, Zambezia, and Cabo Delgado, more of the HIV+ patients were within the age group of 30–34 years.

Most of the participants who tested HIV+ in Nampula and Sofala were in the age groups 40–44 years and 45–49 years, respectively (S1 Table). For education, the majority of the participants who tested HIV+ in Manica, Maputo Cidade, Gaza, Maputo, Zembezia, and Sofala reported no formal education. However, most of the participants who tested HIV+ in Niassa, Nampula, and Cabo Delgado provinces had attained post-secondary education. In Inhambane and Tete provinces, the majority of the participants who tested HIV+ had attained primary and secondary level education, respectively (S1 Table). In all the 11 provinces of Mozambique, the majority of the participants who tested HIV+ reported being ever-married. In 6 out of 11 provinces (Manica, Maputo Cidade, Gaza, Maputo, Zembezia, Sofala), most of the participants who tested positive for HIV identified belonging to the Islamic religion (S1 Table). In Cabo Delgado and Nampula, most of the participants who tested HIV+ reported that they belonged to no religion. While in Niassa and Inhambane province, most of the participants who tested HIV+ identified as protestant Christians (S1 Table).

#### Sexual risk behavioral factors

In Maputo Cidade, Gaza, Cabo Delgado, and Sofala, the majority of the HIV+ participants had had 3–4 total lifetime sexual partners. In Tete, Maputo, Niassa, and Inhambane, most of the participants who tested HIV+ did not disclose their total number of sexual partners. In Nampula and Zembezia, the majority of the HIV+ participants had had 5–10 total lifetime sexual partners, and in Manica province, the majority had had 10+ total lifetime sexual partners (S1 Table). In six out of eleven regions (Tete, Nampula, Zembezia, Niassa, Sofala, and Inhambane), the majority of those who tested HIV+ had used a condom for their most recent sex (S1 Table). In Manica, Maputo Cidade, Gaza, and Maputo, most of the HIV+ participants had not used a condom for their most recent sex (S1 Table). We observed that most of the participants who tested HIV+ in Maputo Cidade, Gaza, Zambezia, Cabo Delgado, Sofala, Inhambane provinces reported to have had an extramarital/union affair with at least one partner (S1 Table).

#### Biological and socioeconomic factors

**Fig 4:**
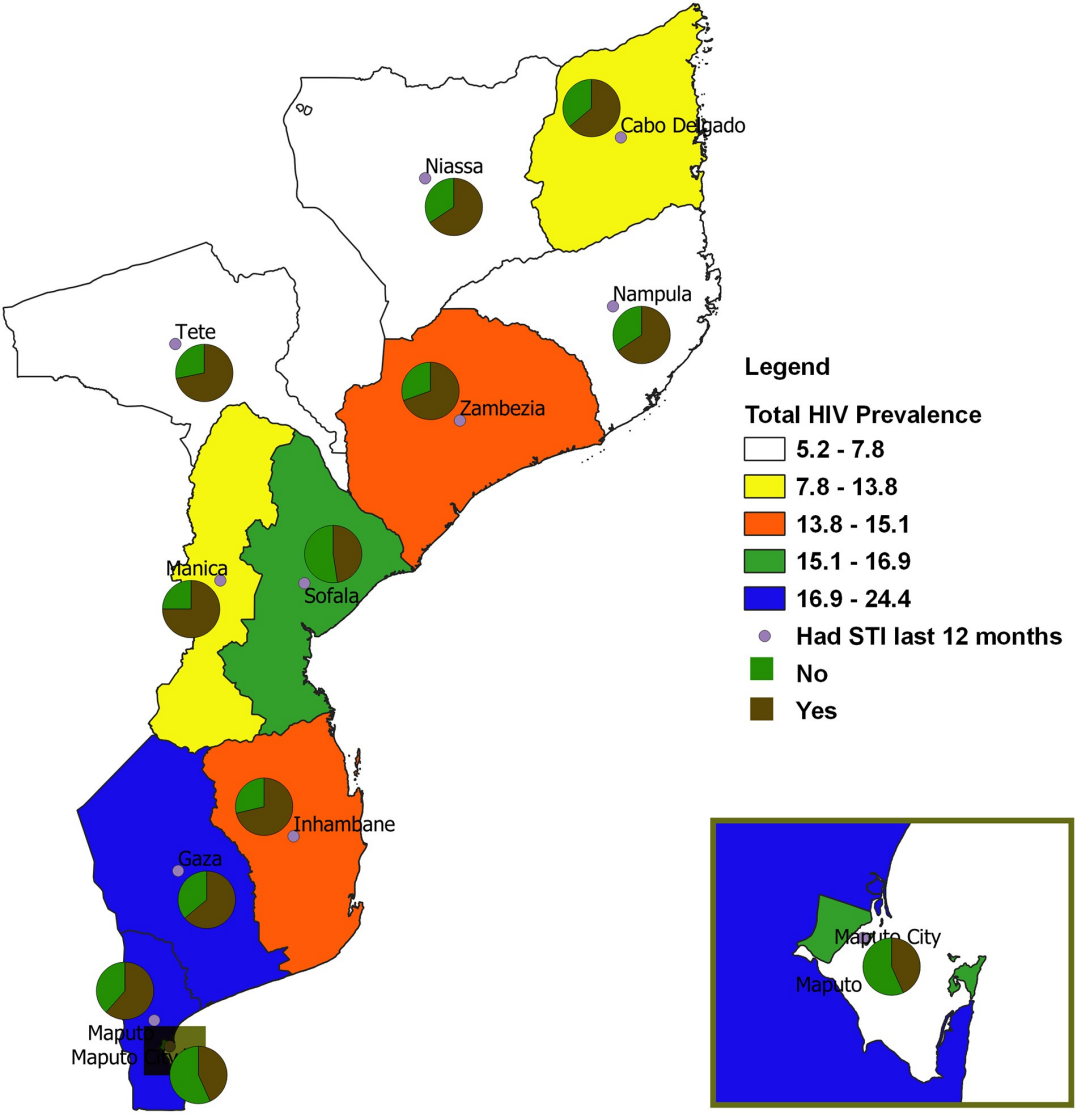
Spatial map of provincial HIV prevalence by any STI last 12 months.

Except for Maputo Cidade and Sofala provinces, the majority of the participants that tested HIV+ in the remaining 9 provinces reported to have had an STI in the 12 months before the AIS survey (Fig 4). We observed that many of the HIV+ respondents in the following provinces were in the richest households: Tete, Manica, Zambezia, Nampula, Niassa, and Safola. However, in Maputo Cidade, Gaza, Maputo, and Inhambane provinces, most of the participants who tested HIV+ reportedly were in poor households (S1 Table).

## Discussion

We modeled the predictors of HIV prevalence in Mozambique through a complex samples logistic regression and spatial mapping approach using nationally representative data. Even though similar studies were previously conducted, to the best of our knowledge this is the first study to use the 2015Mozambique DHS dataset, the most recent available dataset. We found that gender, age, education level, marital status, number of total lifetime sexual partners, history of STI in the last 12 months, urban/rural residence, extramarital sexual affairs, household wealth and region of residence were significant predictors of HIV infection in Mozambique.

Our results showing that females were generally more likely to be infected with HIV compared to males concurs with results of previous studies [7, 27]. Females in many African societies are in vulnerable socioeconomic circumstances, which may force them into risky sexual relationships or behaviors that expose them to a higher risk of HIV infection [27, 28]. These generally documented risks that increased the vulnerability of women to the risk of HIV infection may equally be responsible for our results: poor knowledge about HIV risk, poor access to HIV prevention services due to fear of discrimination by society and heath care workers, gender inequality, gender-based and sexual-based violence has been attributed to the increased vulnerability of women to the risk of HIV infection [29]; however, in our study, there are no variables in the dataset so we could not investigate the possibility of these factors. Also, some cultural practices in Mozambique such as the ritual cleansing for widows could contribute to the spread of HIV among women [9]. For example, in some parts of Mozambique, a widow is required by tradition to undergo a cleansing ritual called “pita-kufa”, which involves several sessions of unprotected sexual intercourse with the brother of her deceased husband. [9] Though our results indicated that females were generally more likely to be infected with HIV, the stratification results indicate that the Nampula region is an exception. This means that the same gender-specific (female targeting) programs that might work in other provinces of Mozambique might not be effective in Nampula.

Empirical evidence on the influence of age with respect to gender and increased HIV incidence reveals that young women aged 15–24 years tend to have higher HIV infection rates, 5–7 years earlier than their male peers [30]. In this study, persons older than 20 years (adults and the elderly) were more likely to test positive for HIV compared to teenagers (15–19 years). Although some young people might engage in risky sexual activities, generally the proportion of adults who engage in sexual activities is higher compared to the teenage population [31]. Therefore, the adult population in Mozambique has a higher probability of testing positive because sexual intercourse is a major medium of HIV/AIDS transmission in the country. Furthermore, being divorced, separated or widowed were shown to be associated with high HIV prevalence in this study, consistent with a study conducted in Uganda that found HIV risk to be higher among currently and previously married individuals than unmarried people [31]. A possible explanation for the high risk of HIV infections among individuals who were ever-married (i.e. those who were divorced, separated and widowed) could be that they may have dissolved their marriages after discovering that they or their partner was HIV-positive, and the widows/widowers may have lost their spouse due to HIV infection. Our results suggest that programs and interventions focusing on control of HIV infection should focus on widowed, divorced and separated individuals and promote appropriate prevention strategies such as condom use, use of post/pre-exposure prophylaxis and abstinence from sexual activities to prevent contracting HIV or other sexually transmitted infections (STIs).

We found that persons coming from a richer household (the fourth wealth quantile on the five-wealth index scale) were more likely to test positive for HIV. This finding may appear to be challenging a long-held belief that lower socioeconomic status is a driver of HIV epidemic. However, our study confirms results of other studies from sub-Saharan Africa using the DHS datasets [32, 33]. This situation may be complex, but it is obvious that there are some poor persons coming from richer households in many sub-Saharan African countries. One of the studies concluded, which we agree with, that “neither poverty nor wealth per se drives the HIV epidemic” in sub-Sharan Africa, and that “being poor or being wealthy may be associated with sets of behaviors that are either protective or risky for HIV infection.”

We found that persons with no education or with primary or secondary education were more likely to test positive for HIV compared with persons with higher formal education. This finding agrees with multiple other studies that found that HIV prevalence is significantly higher among persons with none or lower formal education [4, 20, 34, 35] Possible explanations for this finding could be that people with higher education are more likely to adopt safer service practices which put them at lower risk of contracting HIV compared to less educated people [35]. In addition, people with higher education have higher health seeking behavior compared to those with little none or little formal education [36].

Surprisingly, we found that persons who did not use a condom for their recent sex were less likely to test positive for HIV infection. It is a fact that condom use is protective against HIV infection, so these are the possible explanations to our finding that those who did not use condom in their most recent sex were less likely to test positive for HIV. It is possible that the persons with whom many of these people had recent sexual intercourse with may not be HIV positive. It is also possible some people knew their status as negative and that they had no reason to use a condom especially when they were sure of the negative status of their partners. This finding was consistent with a previous study that found HIV prevalence to be significantly higher among those who reported using a condom at last sex compared to those who reported not using a condom [37], and our explanation could be true in this study, too. We also found that persons who used condom for their most recent sex were more likely to test positive for HIV. It’s likely that many of these persons were aware of their HIV status and may be adhering to the public health advice of adopting the protective measure of using condoms for sex to protect their sexual partners from getting infected.

Our findings also revealed that persons who reported having had an STI in the last 12 months were more likely to be HIV-positive compared to those who had not reported an STI. This finding is consistent with previous studies conducted in Uganda [6], South Africa [7] and Spain [38]. The association between STIs and the risk of HIV infection is well described in the literature. The presence of an STI could be serving as a proxy for unsafe sexual behavior. People who contract STIs are likely to be involved in risky behaviors (i.e. not using condoms and having multiple partners) that increase their chances of being infected with HIV [16, 39].

Our study found that people with more than one lifetime sexual partner were at increased risk of HIV infections. This is consistent with the previous study in Mozambique as part of a study on four Sub-Saharan African countries using 2009 data [23]. Research shows that having multiple sexual partners may be culturally acceptable in Mozambique [8, 40]. This cultural acceptance might encourage many Mozambicans to maintain or frequently change sexual partners, regardless of the risk. Therefore, there is an urgent need for policies to prioritize educational campaigns on social norms and behavioral change to reduce HIV infections attributable to multiple sexual partners.

Our study findings indicate regional variations in HIV prevalence, with southern provinces, especially Gaza and Maputo Provincia, having the highest burdens. This concurs with previous studies that report high HIV prevalence in the southern and central parts of Mozambique [8, 23, 41]. There is evidence suggesting that residents in these parts of the country migrate frequently to high HIV-endemic neighboring countries, such as South Africa, in pursuit of short- and long-term job opportunities [8, 42]. Again, sexual cleaning rituals are more common in these provinces, a ritual that involves unprotected bouts of sexual intercourse [9].

We found that rural residence was protective of HIV infection. This concurs with a previous study conducted in Mozambique that found higher HIV prevalence in urban rather than rural women [43]. HIV infections are also prevalent in regions along the coasts of Mozambique (Zambezia, Sofala, Inhambane, and Maputo Provincia). Fishermen residing along the coast, because of their occupation, are reported to be at a high risk of HIV infection due to their social linkages, mobility to different sites and regional markets, which might contribute a significant quota to the high prevalence reported in these regions. Studies have found that their occupation predisposes them to long periods of staying away from home, high alcohol consumption, and the likelihood of engaging in risky behaviors such as unsafe sex with casual/commercial sex workers [44–46].

### Strengths and limitations

One of the strengths of our study was the use of a large, nationally representative survey data set (2015 Mozambique DHS) that is grounded in standardized methodology for analyses. Secondly, the study employed spatial analytical techniques that have advantages over standard statistical techniques to identify geographical variations of HIV prevalence in Mozambique. Our findings, however, are subject to limitations that must be taken into consideration. As a characteristic of all cross-sectional studies, this study could neither establish temporality nor causality of the observed associations of the predictors with the risk of HIV infection. Secondly, self-reporting of sexual behaviors is prone to recall and social desirability bias. Despite these limitations, this study has provided profound insights from a population-level survey analysis as well as a spatial analysis of HIV prevalence in Mozambique for informed public health action.

## Conclusion

The findings of our study identified gender, age, marital status, the total number of lifetime sexual partners, condom use and diagnosis with other STIs as significant predictors of HIV infection in Mozambique. Furthermore, emphasis on behavioral modification to reduce the risk of HIV infection should be highlighted to help reduce the spread of HIV. The results from the disaggregated analyses visualized through the spatial maps by selected socio-demographic factors, from a program perspective, can be drilled down to even smaller geographic areas such as district and sub-district levels. This would be useful for targeted HIV programming, including testing, linkage to care, treatment support and retention in care as well as preventive programs. These results further suggest that Pre-Exposure Prophylaxis (PrEP) campaigns should be pursued to lower the reservoir of HIV infections among the identified high-risk groups.

## Supporting information

S1 Table Spatial and regional distribution of HIV prevalence (n = 11,270). (DOCX)

## Abbreviations

AIDS: Acquired Immune Deficiency Syndrome
AIS: AIDS Indicator Survey
AOR: Adjusted Odds Ratio
ART: Antiretroviral Therapy
CI: Confidence Interval
DHS: Demographic Health Survey
GPS: Global Positioning System
HIV: Human Immunodeficiency Virus
MDHS: Mozambique Demographic Health Survey
PSU: Primary Sampling Units
QGIS: Quantum Global Positioning System
STI: Sexually Transmitted Infection
WHO: World Health Organization
