# Correction: Predictors of high HIV+ prevalence in Mozambique: A complex samples logistic regression modeling and spatial mapping approaches

**DOI:** 10.1371/journal.pone.0235456

**Published:** 2020-06-25

**Authors:** The PLOS ONE Staff

The first author’s initials appear incorrectly in the citation. The publisher apologizes for the error. The correct citation is: Nutor JJ, Duodu PA, Agbadi P, Duah HO, Oladimeji KE, Gondwe KW (2020) Predictors of high HIV+ prevalence in Mozambique: A complex samples logistic regression modeling and spatial mapping approaches. PLoS ONE 15(6): e0234034. https://doi.org/10.1371/journal.pone.0234034.
