# Supplementary material for: Predictors of high HIV+ prevalence in Mozambique: A complex samples logistic regression modeling and spatial mapping approaches
Source: PLoS One. 2020 Jun 4;15(6):e0234034. doi: 10.1371/journal.pone.0234034 (PMC7272061; doi:10.1371/journal.pone.0234034)
Supplement: S1 Table — (DOCX) [file pone.0234034.s001.docx]

**S1 Table: Spatial and Regional Distribution of HIV Prevalence (n= 11,270)**

|  | **Region** | | | | | | | | | | |
| --- | --- | --- | --- | --- | --- | --- | --- | --- | --- | --- | --- |
|  | **Niassa** | **Cabo Delgado** | **Nampula** | **Zambezia** | **Tete** | **Manica** | **Sofala** | **Inhambane** | **Gaza** | **Maputo** | **Maputo City** |
| **Gender** |  |  |  |  |  |  |  |  |  |  |  |
| Male | 4.40 | 10.60 | 6.20 | 12.70 | 3.20 | 11.10 | 12.70 | 8.00 | 18.80 | 16.70 | 12.10 |
| Female | 9.80 | 15.70 | 5.10 | 16.20 | 6.10 | 15.20 | 18.40 | 16.10 | 28.50 | 28.00 | 21.70 |
| **Age group** |  |  |  |  |  |  |  |  |  |  |  |
| 15-19 yrs | 2.40 | 9.30 | 2.50 | 8.30 | 0.80 | 3.70 | 4.00 | 3.50 | 3.40 | 2.60 | 4.50 |
| 20-24 yrs | 3.60 | 10.20 | 5.60 | 13.40 | 1.90 | 11.80 | 10.00 | 14.20 | 21.20 | 14.50 | 10.20 |
| 25-29 yrs | 6.60 | 19.30 | 7.00 | 14.50 | 5.80 | 13.80 | 18.90 | 18.50 | 36.40 | 23.50 | 20.00 |
| 30-34 yrs | 9.40 | 22.80 | 7.00 | 23.50 | 3.50 | 17.30 | 21.40 | 20.50 | 39.40 | 34.90 | 28.60 |
| 35-39 yrs | 13.70 | 10.80 | 5.70 | 19.50 | 13.70 | 28.60 | 32.00 | 29.20 | 39.30 | 53.00 | 34.50 |
| 40-44 yrs | 11.50 | 21.00 | 7.10 | 16.90 | 9.10 | 20.80 | 18.20 | 17.20 | 33.30 | 39.60 | 36.70 |
| 45-49 yrs | 19.00 | 9.70 | 6.60 | 14.30 | 6.80 | 15.60 | 32.40 | 12.70 | 31.70 | 32.40 | 17.00 |
| 50-54 yrs | 4.50 | 11.50 | 4.40 | 9.10 | 0.00 | 20.60 | 17.30 | 10.20 | 37.10 | 24.40 | 29.50 |
| 55+ yrs | 3.40 | 8.50 | 5.00 | 18.40 | 7.40 | 4.30 | 11.80 | 3.80 | 20.50 | 17.10 | 14.30 |
| **Level of education** |  |  |  |  |  |  |  |  |  |  |  |
| No education | 5.60 | 11.50 | 6.40 | 14.70 | 5.40 | 15.50 | 20.50 | 15.80 | 33.60 | 31.90 | 38.50 |
| Primary | 6.80 | 12.40 | 4.40 | 13.00 | 3.70 | 12.70 | 14.50 | 16.60 | 27.70 | 31.60 | 27.10 |
| Secondary | 10.60 | 17.30 | 8.10 | 22.90 | 10.60 | 13.40 | 15.60 | 5.90 | 16.20 | 12.90 | 11.80 |
| Post-secondary | 25.50 | 17.60 | 9.50 | 8.30 | 0.00 | 7.70 | 6.70 | 0.00 | 8.30 | 4.00 | 2.90 |
| **Marital status** |  |  |  |  |  |  |  |  |  |  |  |
| Never in union | 2.80 | 8.60 | 4.70 | 11.90 | 1.80 | 4.70 | 5.30 | 7.90 | 11.40 | 9.20 | 4.60 |
| Married | 6.90 | 14.30 | 5.80 | 13.00 | 5.00 | 14.60 | 15.90 | 11.20 | 9.40 | 18.30 | 15.50 |
| Living with partner | 8.70 | 12.00 | 2.90 | 11.50 | 3.90 | 15.50 | 13.60 | 15.00 | 26.20 | 26.20 | 22.30 |
| Widowed | 23.10 | 24.00 | 2.00 | 23.80 | 20.00 | 23.50 | 38.20 | 26.70 | 49.40 | 52.00 | 60.00 |
| Divorced | 6.30 | 27.60 | 9.50 | 28.60 | 10.70 | 41.70 | 47.10 | 12.50 | 0.00 | 50.00 | 14.30 |
| No longer living together/separated | 7.90 | 15.30 | 9.20 | 25.90 | 10.30 | 22.00 | 39.60 | 22.70 | 47.60 | 47.80 | 38.90 |
| **Religion** |  |  |  |  |  |  |  |  |  |  |  |
| No religion | 0.00 | 19.60 | 9.70 | 16.00 | 4.90 | 11.00 | 16.10 | 10.00 | 26.50 | 21.40 | 15.80 |
| Catholic | 8.20 | 18.60 | 6.00 | 13.80 | 2.40 | 19.00 | 14.10 | 5.80 | 21.40 | 22.50 | 10.40 |
| Protestants | 9.50 | 10.30 | 3.00 | 12.40 | 7.00 | 13.90 | 16.40 | 15.70 | 25.60 | 22.80 | 19.70 |
| Islam | 6.60 | 9.50 | 5.20 | 26.50 | 0.00 | 15.80 | 22.70 | 6.70 | 27.30 | 23.50 | 19.50 |
| **Total lifetime sexual partner** | | |  |  |  |  |  |  |  |  |  |
| 0 | 0.00 | 0.00 | 3.80 | 1.60 | 0.00 | 2.90 | 0.00 | 2.70 | 1.20 | 0.00 | 2.80 |
| 1 | 3.40 | 8.80 | 3.50 | 7.20 | 4.40 | 11.80 | 13.50 | 6.70 | 13.90 | 13.10 | 8.50 |
| 2 | 11.10 | 10.70 | 6.40 | 16.00 | 7.00 | 19.60 | 19.70 | 17.20 | 31.20 | 24.60 | 18.40 |
| 3-4 | 12.20 | 16.40 | 6.20 | 24.00 | 5.50 | 16.70 | 25.30 | 13.60 | 36.40 | 29.90 | 23.70 |
| 5-9 | 1.70 | 23.00 | 8.50 | 24.80 | 8.80 | 25.00 | 17.50 | 17.90 | 31.40 | 23.00 | 22.10 |
| 10+ | 2.80 | 10.00 | 2.40 | 13.30 | 0.00 | 28.60 | 23.70 | 11.50 | 31.80 | 22.80 | 18.60 |
| Undisclosed | 16.70 | 8.40 | 5.60 | 18.80 | 9.10 | 20.00 | 18.60 | 23.60 | 29.40 | 43.80 | 22.30 |
| **Number of extra marital affairs** | | |  |  |  |  |  |  |  |  |  |
| 0 | 7.50 | 13.00 | 5.20 | 21.50 | 4.90 | 14.10 | 15.90 | 12.90 | 24.10 | 23.70 | 18.00 |
| 1 | 5.70 | 16.70 | 7.70 | 21.90 | 5.80 | 9.00 | 16.70 | 14.70 | 27.80 | 22.80 | 17.20 |
| 2+ | 12.50 | 6.30 | 12.50 | 9.80 | 0.00 | 21.40 | 12.00 | 0.00 | 23.30 | 14.80 | 14.10 |
| **Had STI last 12 months** | | |  |  |  |  |  |  |  |  |  |
| NO | 7.10 | 12.70 | 5.50 | 13.80 | 4.90 | 12.60 | 16.20 | 12.70 | 24.40 | 22.00 | 17.70 |
| YES | 13.60 | 22.40 | 10.50 | 31.50 | 12.50 | 37.90 | 14.70 | 31.60 | 43.20 | 35.10 | 13.50 |
| **Used condom for most recent sex** | | |  |  |  |  |  |  |  |  |  |
| No | 7.10 | 13.40 | 5.60 | 14.00 | 4.00 | 13.90 | 15.20 | 15.00 | 27.20 | 24.00 | 18.90 |
| Yes | 12.80 | 10.10 | 10.70 | 18.20 | 8.40 | 10.60 | 18.90 | 10.40 | 26.00 | 22.20 | 17.00 |
| Never had sex | 0.00 | 0.00 | 3.80 | 1.60 | 0.00 | 2.90 | 0.00 | 2.70 | 1.20 | 0.00 | 2.80 |
| Not had sex last month | 9.30 | 18.70 | 5.20 | 21.30 | 9.90 | 23.60 | 26.20 | 16.70 | 33.00 | 33.90 | 25.60 |
| **Household wealth** |  |  |  |  |  |  |  |  |  |  |  |
| Poorest | 3.50 | 12.60 | 5.60 | 13.00 | 5.40 | 22.00 | 19.50 | 18.90 | 20.00 | 0.00 | 0.00 |
| Poorer | 4.90 | 11.60 | 3.90 | 11.80 | 4.60 | 8.10 | 11.60 | 13.00 | 43.20 | 33.30 | 0.00 |
| Middle | 8.70 | 11.60 | 6.10 | 13.90 | 1.60 | 8.20 | 12.60 | 19.10 | 25.30 | 37.80 | 0.00 |
| Richer | 12.90 | 21.20 | 6.60 | 23.20 | 4.00 | 17.80 | 22.20 | 12.30 | 27.00 | 27.70 | 34.70 |
| Richest | 18.00 | 9.80 | 7.90 | 24.20 | 22.00 | 15.90 | 19.30 | 6.10 | 19.80 | 19.90 | 16.30 |
| **Urban/rural residence** | | |  |  |  |  |  |  |  |  |  |
| Urban | 13.80 | 15.60 | 7.80 | 23.10 | 14.00 | 17.90 | 21.20 | 14.80 | 23.00 | 21.50 | 17.40 |
| Rural | 5.50 | 12.40 | 4.60 | 12.20 | 3.40 | 11.10 | 12.70 | 12.60 | 26.10 | 26.50 | 17.40 |
